# Activated iNKT cells enhance the anti-tumor effect of antigen specific CD8 T cells on mesothelin-expressing salivary gland cancer

**DOI:** 10.1186/s12885-018-5179-7

**Published:** 2018-12-17

**Authors:** Yuji Makita, Naoki Kunii, Daiju Sakurai, Fumie Ihara, Shinichiro Motohashi, Akane Suzuki, Toshinori Nakayama, Yoshitaka Okamoto

**Affiliations:** 1 Department of Otorhinolaryngology, Head and Neck Surgery, Graduate School of Medicine, Chiba University, 1-8-1 Inohana, Chuo-ku, Chiba, 260-8670 Japan; 2 Department of Medical Immunology, Graduate School of Medicine, Chiba University, Chiba, Japan; 3 Department of Immunology, Graduate School of Medicine, Chiba University, Chiba, Japan

**Keywords:** Adoptive immunotherapy, Chimeric antigen receptor, Cytotoxic T lymphocyte, Natural kiiler T-cells, Salivary gland cancer

## Abstract

**Background:**

Salivary gland cancers are not sensitive to conventional radiotherapy or chemotherapy regimens. Therefore, the development of a new treatment strategy is of critical importance for improving the prognosis. We examined the expression of mesothelin molecules in salivary gland cancers and the efficacy of adoptive cell therapy based on mesothelin-specific chimeric antigen receptor transduced T cells.

**Methods:**

The expression of mesothelin molecule was studied in salivary gland cancer samples obtained from 16 patients as well as a salivary gland cancer cell line (A-253) and five other cell lines. The activation of mesothelin-specific chimeric antigen receptor-expressing CD8 T cells after stimulation with mesothelin and the effects of invariant natural killer T cells on this activation were evaluated.

**Results:**

Mesothelin was detected in the A-253 cells and the surgical specimens except for the case of squamous cell carcinoma to various degrees. Following stimulation with mesothelin expressing cancer cells, chimeric antigen receptor T cells were dose-dependently activated; this activation was enhanced by co-culture with invariant natural killer T cells and subsequently abrogated by treatment with anti-interferon-γ antibodies. Furthermore, the cytotoxicity of chimeric antigen receptor T cells against various cancer cells was further augmented by invariant natural killer T cells.

**Conclusions:**

The use of adoptive transfer with mesothelin-specific chimeric antigen receptor-expressing CD8 T cells against salivary gland cancers is an effective therapy and invariant natural killer T cells are expected to be used in adjuvant treatment for T cell-based immunotherapy.

**Electronic supplementary material:**

The online version of this article (10.1186/s12885-018-5179-7) contains supplementary material, which is available to authorized users.

## Background

Salivary grand cancers (SGCs) exhibit a broad-spectrum of phenotypic, biological and clinical diversity [1, 2]. High-grade malignancies of SGCs (e.g., mucoepidermoid carcinoma (high-grade type), adenoid cystic carcinoma, salivary duct carcinoma and carcinoma ex pleomorphic adenoma, etc.) carry a poorer prognosis [3, 4]. The first choice of clinical treatment for resectable SGC is surgical excision [5], and adjuvant radiation therapy has the potential to increase survival [6, 7]. However, the sensitivity of most SGCs to conventional radiation therapy and chemotherapy regimens is not sufficiently certified [8]. Recently, the novel approach of radiation therapy such as intensity modulated radiation therapy (IMRT), accelerated hyperfractionated photon-beam therapy were developed to improve the local control of unresectable and recurrent salivary gland tumors [9–11]. However, the adverse events associated with these therapies have not been fully evaluated.

Chimeric antigen receptors (CARs) are recombinant receptors with the characteristics of antibody-based specificity and the ability to trigger T cell activation [12–15]. Transduced CARs provide T cells with the properties of antigen-specific recognition, activation and proliferation, independent of their major histocompatibility complex (MHC) [12, 16, 17], and adoptive cellular therapy using redirected T cells with CARs is a promising immunotherapeutic strategy [18, 19]. However, the tumor-specific antigens in most cancers are not yet well defined [20], and it is thus critical to identify adequate target antigens when applying CAR-based immunotherapy clinically. One attractive tumor target is mesothelin (MSLN), a membranous glycoprotein expressed in a variety of cancers, including mesothelioma, ovarian cancer and pancreatic cancer [21–24]. MSLN-specific CARs that consist of a MSLN-specific single chain variable fragment (SS1-scFv) linked to the CD3ζ signaling molecule with co-stimulatory molecules, such as CD28, CD137 (4-1BB) or CD278 (inducible T cell co-stimulator, ICOS), was recently produced and a clinical study of its effectiveness is ongoing [25]. Although there have been a few reports of the eradication of solid tumors with CAR-expressing T cells [26], solid tumors appear to be a less effective target for CAR-expressing T cells than hematological malignancies [27]. In order to apply immunotherapy regimens using MSLN-specific CAR T cells in cases of SGC, it may be necessary to develop adjuvant agents that enhance the anti-tumor activity.

Invariant natural killer T (iNKT) cells have invariant antigen receptors that recognize glycolipid antigens, such as α-galactosylceramide (αGalCer), presented by CD1d molecules [28–32]. Following activation, iNKT cells exert cytotoxic effects on a variety of cancer cells and we previously showed that activated iNKT cells and αGalCer-loaded dendritic cells (DCs) reduce the tumor volume in patients with head and neck squamous cell carcinoma (HNSCC) in clinical studies [33–36]. It has been reported that large amount of interferon-γ (IFNγ) produced by iNKT cells induce the activation of other effector cells, such as natural killer (NK) cells and cytotoxic T lymphocytes (CTLs), and these effector cells in tumor site play an important role in the expression of the anti-cancer effects [37, 38]. However, the experiments about these activation mechanisms between iNKT cells and CTLs had not been performed because it was difficult to prepare the antigen specific T lymphocyte at high purity.

In this report, we confirmed that most of SGC specimens expressed MSLN molecule. In addition, we demonstrate that the IFNγ produced by iNKT cells augments the cytotoxicity of CTLs against MSLN-expressing cancer cells using MSLN-specific CAR T cells, and moreover discussed the feasibility of adoptive cell therapy for SGC using the combination of MSLN-specific CAR-expressing T cells and iNKT cells.

## Methods

### Construction of the anti-MSLN CAR and vector production

The pELNs vector-based third-generation MSLN-targeted CAR, constructed using MSLN-specific SS1-scFv with or without CD28, 4-1BB and the CD3ζ signaling domains (SS1–28-BB-ζ or SS1-Δζ) were transfected into 293 T cells using FuGENE HD reagent according to the manufacturer’s protocol (Promega, Madison, WI) [25]. The method for generating the lentivirus supernatant has been described previously [39].

### Cell lines

A-253 cells (HTB-41™) derived from epidermoid carcinoma of the submaxillary salivary gland and FaDu cells (HTB-43™) derived from hypopharyngeal squamous cell carcinoma (SCC) were purchased from ATCC (Manassas, VA), and IMC-3 cells derived from maxillary sinus SCC were kindly provided by Dr. N. Seki, Chiba University. M108 cells derived from the malignant pleural effusion of a mesothelioma patient and MSLN-transduced K562 (K562-MSLN) cells were kindly provided by Dr. C. H. June, University of Pennsylvania [25]. The A-253 cells were maintained with McCoy’s 5a Medium (ATCC) containing 10% fetal bovine serum (FBS). The IMC-3 cells and K562-MSLN cells were cultured in RPMI 1640 (Life Technologies Japan, Tokyo, Japan) with 10% FBS and the FaDu cells were subcultured in DMEM (Life Technologies Japan) with 10% FBS.

### Preparation of iNKT cells and CAR-transduced CD8 T cells

iNKT cells of high purity were generated as previously described [34, 40]. Briefly, peripheral mononuclear cells (PBMCs) were separated via density gradient centrifugation using Ficoll-Paque PLUS (GE Healthcare Life Science, Pittsburgh, PA) and cultured in the presence of 100 U/mL of recombinant human IL-2 (rhIL-2, Imunace; Shionogi, Osaka, Japan) and 100 ng/mL of αGalCer (REGiMMUNE, Tokyo, Japan). After seven days of culture, the Vα24-positive iNKT cells were purified using the MACS separation system (Miltenyi Biotec, Bergisch Gladbach, Germany) and incubated for an additional seven days.

CD8 T cells were isolated from the peripheral blood via negative selection using RosetteSep Human CD8 T Cell Enrichment Cocktail (STEMCELL Technologies INC., Vancouver, Canada) and stimulated with Dynabeads Human T-Activator CD3/CD28 (Life Technologies Japan) at a bead-to-cell ratio of 1:1 in medium supplemented with 300 U/mL of rhIL-2. Lentiviral vector supernatant of SS1-Δζ or SS1–28-BB-ζ was added at 24 h after the initial stimulation. The CAR T cells were expanded for 10 days and subsequently used for the experiments.

All specimens were collected according to the Chiba University Institutional Review Board-approved protocol (No.1016), and written informed consent was obtained from each donor.

### Flow cytometric analysis

In order to detect iNKT cells and CAR-expressing CD8 T cells, the following antibodies were used: anti-Vα24, Vβ11 (Beckman Coulter, Brea, CA), anti-CD8 (BD Biosciences), biotin-labeled polyclonal goat anti-mouse F(ab)2 antibodies (Jackson ImmunoResearch, West Grove, PA), streptavidin-APC (BD Biosciences) and anti-human MSLN (R&D Systems, Minneapolis, MN). Dead cells were stained with 7-aminoactinomycin D (7AAD; BD Bioscience). The fluorescence intensity was measured using the FACSCanto II instrument (BD Bioscience), and the data were analyzed with the FlowJo software program (Tree Star, Ashland, OR).

### Tissue preparation, immunohistochemistry and quantitative RT-PCR

Tissue samples were obtained from surgical specimens harvested from patients with salivary gland tumor who underwent tumor resection at Chiba University Hospital, Chiba, Japan. The study protocol was approved by the Institutional Ethics Committee (no. 1336) and conformed to the provisions of the Declaration of Helsinki, 1995. In addition, written informed consent was obtained from all patients prior to surgery.

Tissue fixation and preparation and staining of the tissue sections were performed as previously described [41]. Anti-human mesothelin mouse monoclonal antibodies (Novocastra clone 5B2; Leica, Buffalo Grove, IL), Alexa Flour 594-conjugated goat anti-mouse IgG (Life Technologies Japan) and DAPI (Dojindo, Kumamoto, Japan) were used for staining. All histological analyses were carried out using a confocal laser microscope (LSM710; Carl Zeiss, Oberkochen, Germany). MSLN expressing cells were counted by fluorescence microscopy and classified into 5 groups (−, stained cells < 5%; 1+, 5–20%; 2+, 20–50%; 3+, 50–70%; 4+, > 70%). The tumor tissues of an oncocytoma and pleomorphic adenoma were used as positive and negative control. One well-experienced pathologist, who acted completely independently of this study, histologically diagnosed the tissues.

The relative expression of MSLN were measured by quantitative RT-PCR as described previously [35]. Primers specific for the constant region of the MSLN (sense, GAATGTGAGCATGGACTTGG; antisense, ACGTGGGGTCCCAGAAGT) were used to detect the MSLN molecule of the surgical specimens. All quantitative RT-PCR studies were performed using the Lightcycler 480 II instrument (Roche Applied Science, Indianapolis, IN). The expression was normalized using the GAPDH.

### CD107a mobilization assay

The CD107a mobilization assay was performed as previously described [42]. Briefly, 1 × 10^6^ of M108, A253, IMC3 and FaDu target cells were incubated in each well of a 96-well flat bottom plate overnight in order to adhere the cells to the bottom of the plate. K562-MSLN cells were also incubated overnight. A total of 1 × 10^6^ CAR T cells with or without 3 × 10^6^ of iNKT cells and αGalCer-loaded antigen-presenting cells (APCs) and 10 μL of CD107a-PE antibodies (BD Biosciences) were added to each well. In order to turn off the effects of IFNγ, 5 μg of anti-human IFNγ antibodies (BioLegend, San Diego, CA) was added. Two microliters of monensin (eBioscience) was spiked and the cells were collected after 3.5 h of incubation. Finally, the cells were stained with anti-mouse IgG, Vα24, streptavidin, CD8 and 7AAD and analyzed using flow cytometry.

### Cellular proliferation assay

The cellular proliferation assay was performed as previously described [42]. Briefly, CAR T cells were stained with PKH26 dye (Sigma-Aldrich, St. Louis, MO) according to the manufacturer’s protocol. CAR T cells, iNKT cells and αGalCer-pulsed APCs treated with or without anti-IFNγ were supplemented with 1 × 10^6^ of A-253, IMC-3, FaDu and K562-MSLN cells. After 72 h of incubation, the cells were collected and stained with anti-mouse IgG, anti-Vα24, anti-CD8 and 7AAD. The fluorescence intensity of PKH26 in the CAR T cells was evaluated using flow cytometry and T(X) between 0 and 72 h of CAR T cells was calculated using population comparison mode in FlowJo software as previously published [43, 44].

### In vitro cytotoxicity assay

A-253, IMC-3, FaDu and K562-MSLN cells were incubated with 100 of μCi sodium chromate (PerkinElmer, Waltham, MA) for 1 h. The CAR T cells, iNKT cells or 1 to 1 mixtures of CAR T cells and iNKT cells treated with irradiated αGalCer-loaded APCs were added to ^51^Cr-labeled target cells (1 × 10^4^) at several E: T ratios. After four hours of incubation, the supernatant was collected and the dose of radiation was measured using a Packard COBRA II Automated Gamma Counter (GMI Inc., Ramsay, MN).

### Statistical analysis

Student’s *t*-test was used to evaluate the significance of differences between the two groups of homoscedastic samples.

## Results

### Expression of MSLN molecules in the salivary gland cancer cells

**Fig. 1:**
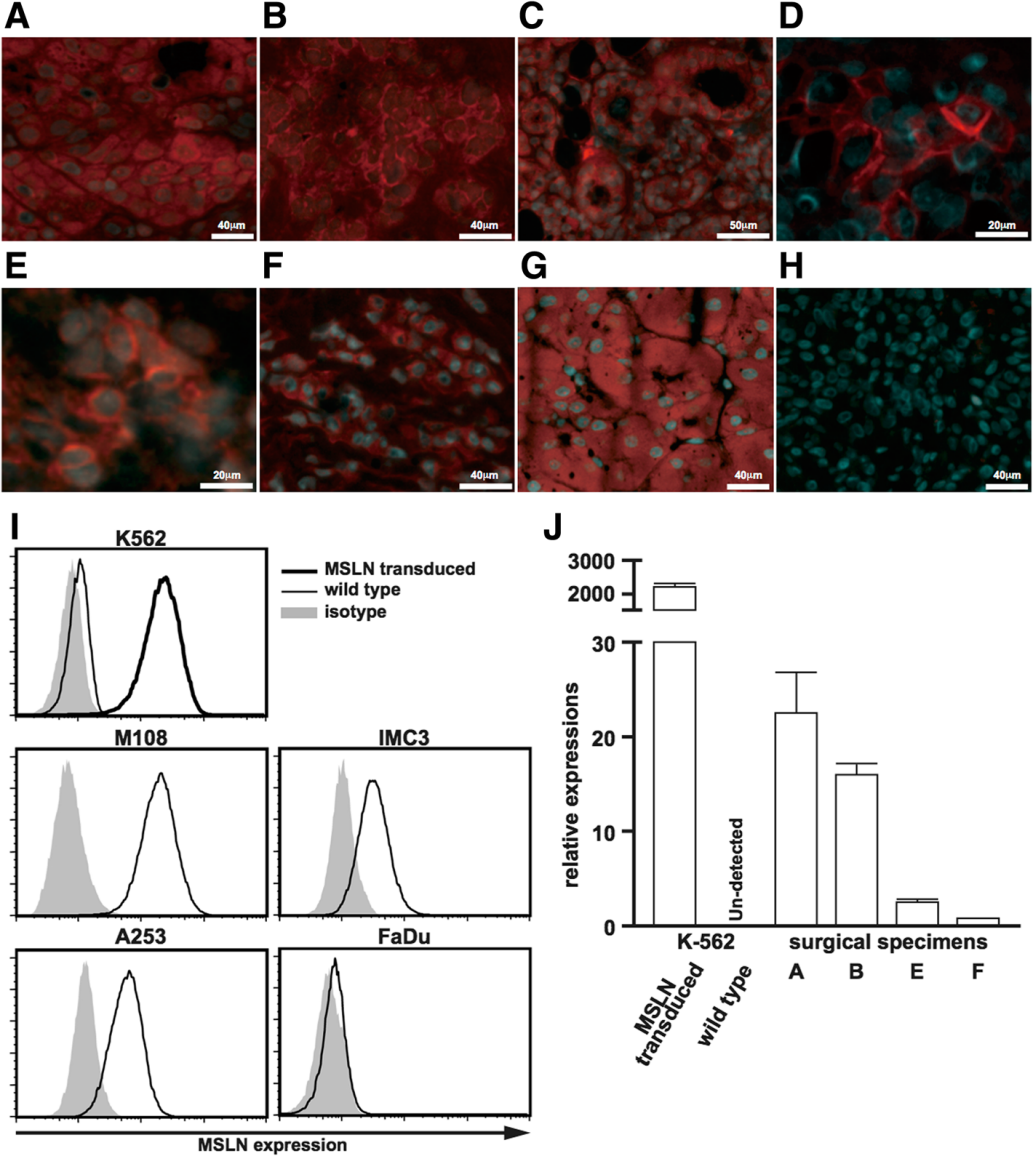
MSLN expressions in the surgical samples obtained from the patients with various types of SGC and the various cancer cell lines. **a**-**h** Immunohistochemical images of mucoepidermoid carcinoma (a, case 12 and d, case 5), adenocarcinoma (b, case 13), salivary duct carcinoma (c, case 16 and f, case 15) and adenoid cystic carcinoma (e, case 11) are shown. Positive control tumor (**g**) and negative control pleomorphic adenoma (h, case 17) are also shown. The red and blue colors indicate MSLN and DAPI. **i** Histograms of MSLN expression on the various cancer cell lines analyzed by flow cytometry are shown. Gray shadows indicate isotype matched control. **J** RT-PCR analysis of MSLN were performed with case 12 (**a**), case 13 (**b**), case 11 (**e**) and case 15 (**f**). The MSLN-transduced or wild type K-562 cells were used for positive and negative control

First of all, we examined the tumor tissues to evaluate the proportions of MSLN-expressing cells. The surgical specimens of SGCs except for the case of squamous cell carcinoma expressed MSLN in a wide variety of the intensity (Table 1. and Fig. 1a-f). The mucoepidermoid carcinoma (Fig. 1a and d), the adenoid cystic carcinoma (Fig. 1c and e), the non-specific adenocarcinoma (Fig. 1b) and some of the salivary duct carcinoma samples (Fig. 1f) included more MSLN positive cells in the cancer tissues. However, the expression levels in the surgical specimens were highly variable even in the same specimen (Fig. 1c and f). The MSLN-expressing oncocytoma specimen that were benign epithelial tumor was utilized as positive control (Fig. 1g). MSLN-expressing cell was not detected in the pleomorphic adenoma that is the most frequent benign neoplasm in the salivary glands (Fig. 1h).

**Table 1:** MSLN expressions in the surgical specimens with various pathological diagnosis of SGC

Pathological diagnosis	Mesothelin expression^a^	Case
Salivary duct carcinoma	3+	1
Salivary duct carcinoma	1+	3
Salivary duct carcinoma	1+	4
Salivary duct carcinoma	3+	15
Salivary duct carcinoma	3+	16
Mucoepidermoid carcinoma	3+	5
Mucoepidermoid carcinoma	2+	7
Mucoepidermoid carcinoma	3+	9
Mucoepidermoid carcinoma	4+	12
Adenoid cystic carcinoma	3+	6
Adenoid cystic carcinoma	4+	11
Adenocarcinoma	4+	13
Adenocarcinoma	2+	14
Acinic cell carcinoma	–	2
Acinic cell carcinoma	2+	10
Squamous cell carcinoma	–	8
Pleomorphic adenoma	–	17

^a^–, mesothelin^+^ cells < 5%; 1+, 5–20%; 2+, 20–50%; 3+, 50–70%; 4+, > 70%

In the study with the several cell lines, MSLN was mildly expressed in the A-253 cells compared with the K562-MSLN cells and M108 cells those were positive controls, whereas the expression level in the A-253 cells was remarkably higher than that observed in the IMC-3 cells (derived from human maxillary squamous cell carcinoma) or FaDu cells (derived from human pharyngeal squamous cell carcinoma) (Fig. 1i and Additional file 1: Figure S1).

Moreover, the relative expressions (MSLN/GAPDH) of these surgical specimens (Fig. 1a, b, e and f) were measured by RT-PCR and MSLN expressions with wide variations were confirmed (Fig. 1j). The MSLN-transduced or wild type K-562 cells were used for positive and negative control.

### Preparation of MSLN-specific CAR redirected CD8 T cells and highly purified iNKT cells from primary human PBMCs

**Fig. 2:**
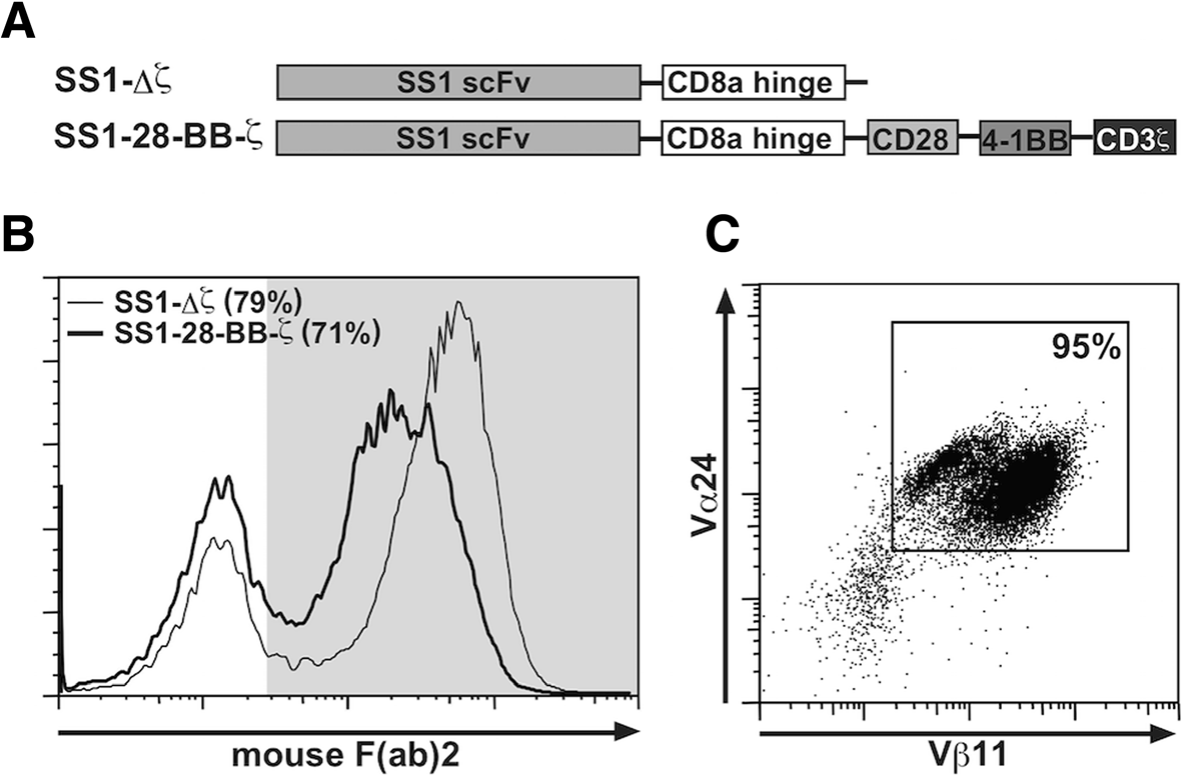
Preparation of the anti-MSLN lentiviral vector engineered with CD8 T cells and highly purified iNKT cells. **a** Schematic representation of MSLN-binding CARs. For a binding-control CAR, a truncated TCRζ domain was utilized. **b** The expression levels of the SS1 scFv fusion proteins on human primary CD8 T cells were examined using flow cytometry. The thin and bold lines indicate SS1-Δζ and SS1–28-BBζ, respectively. **c** iNKT cells were purified from human PBMCs stimulated with αGalCer for eight days

SS1 scFv recognizes human MSLN with high binding affinity (Kd = 0.7 nM) [45]. Two types of SS1 scFv-based CARs with or without the CD3ζ signal-transduction domain and CD28/CD137 (4-1BB) intracellular domains in tandem (SS1–28-BB-ζ or SS1-Δζ) (Fig. 2a) were lentivirally transduced into purified primary human CD8 T cells. SS1-Δζ CAR expressing CD8 T cells were used for negative control. The transduction efficacy of these cells was analyzed using anti-mouse F(ab)2 antibodies on the eighth day after stimulation. The proportion of CAR-expressing cells was 71% in the SS1–28-BB-ζ cells and 79% in the SS1-Δζ cells (Fig. 2b). iNKT cells in PBMCs were expanded via αGalCer stimulation and purified to reach a proportion of more than 95% (Fig. 2c).

### The activation of CD8 CAR T cells was induced by the recognition of MSLN molecules on the surface of the SGC cells and augmented by the presence of iNKT cells

**Fig. 3:**
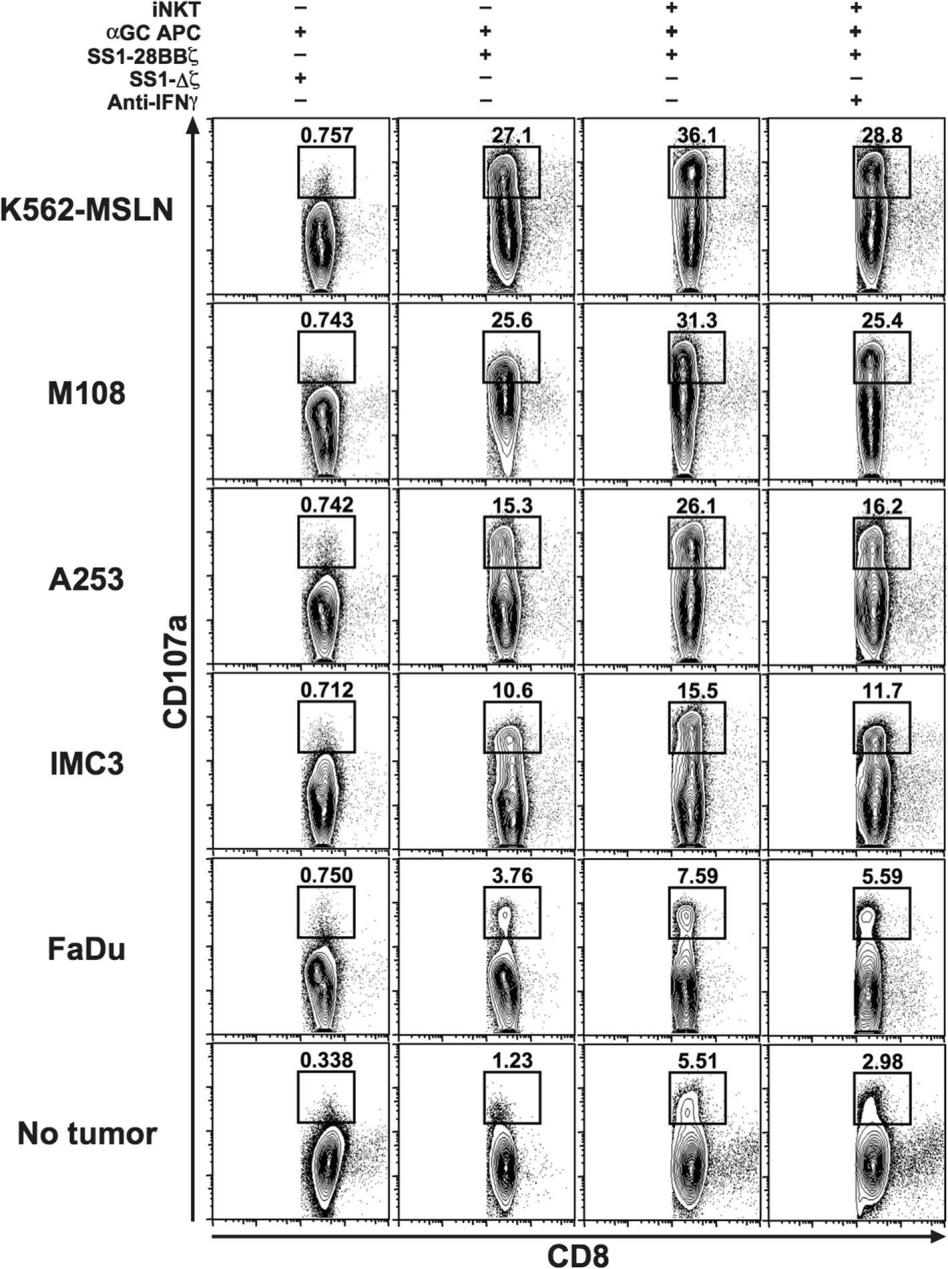
CD107a mobilization assay of SS1 CAR-expressing human CD8 T cells after stimulation of the various cancer cell lines with or without the adjuvant activated iNKT cells. The engineered T cells were incubated for 3.5 h after stimulation and analyzed. CD8^+^CAR^+^7AAD^−^ cells are shown

The stimulation of SS1 CAR-expressing CD8 T cells by various tumor cell lines including A-253 did not increase the translocation of CD107a on the surface of the SS1-Δζ transduced T cells because these CAR T cells could not transduce the activation signal after antigenic recognition (Fig. 3, first column). The CD107a mobilization of the SS1–28-BB-ζ transduced cells was clearly induced by the stimulation with tumor cell lines used. The intensities of CD107 seemed to be dependent on the degree of the MSLN expression (Fig. 3, second column). iNKT cells increased the translocation of CD107a on the SS1–28-BB-ζ transduced cells, namely, the activation of these CAR T cells were augmented by the addition of iNKT cells compared with CAR T cells alone exposed to any types of cell lines. (Fig. 3, third column). iNKT cells could induce the activation of CAR T cells in some degree even in the absence of tumor cells (Fig. 3, no tumor). These enhanced activations by iNKT cells were blocked by the treatment with IFNγ blockade (Fig. 3, fourth column).

### Enhanced proliferation of CAR T cells after stimulation of the SGC cells by activated iNKT cells

**Fig. 4:**
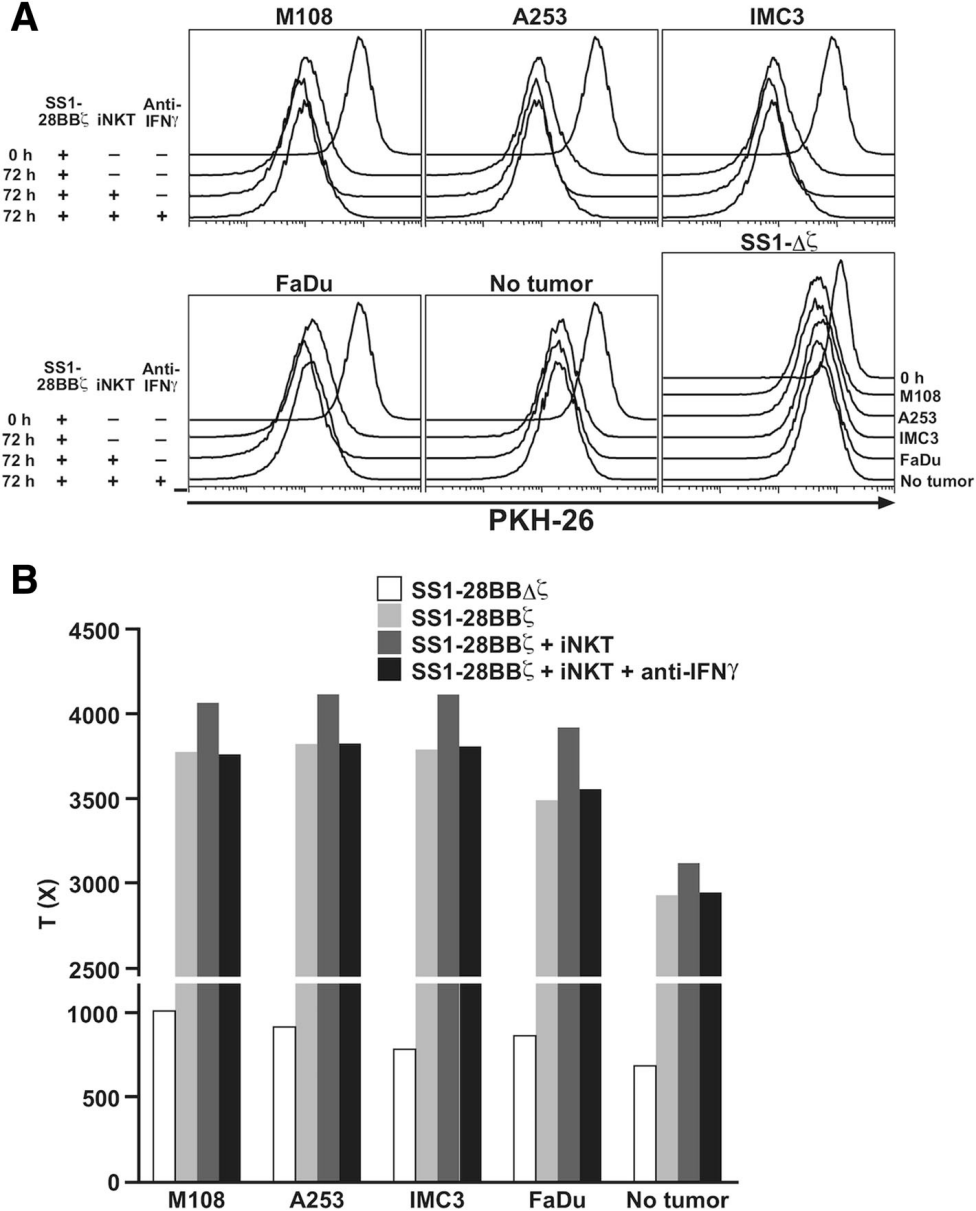
Cellular proliferation of SS-1 CAR-transduced CD8 T cells. CAR-expressing CD8 T cells were stained with PKH26 and cultured for 72 h in the presence of M108, A-253, IMC3 and FaDu tumor cells. **a** A histogram of the PKH26 expression of CD8^+^CAR^+^Vα24^−^7AAD^−^ cells and (**b**) proliferative index (T(x)) are shown

The proliferation of SS1-Δζ-expressing T cells was very low even after stimulation with the tumor cells expressing high levels of MSLN (Fig. 4 a, lower right column and B, open bar). On the other hand, the significant expansion of SS1–28-BB-ζ T cells was detected after the similar stimulation (Fig. 4 b, light gray bar) and the degree was elevated by the co-culture with iNKT cells (Fig. 4 b, dark gray bar), Moreover, these amplifying effects by iNKT cells were completely blocked by the treatment with anti-IFNγ (Fig. 4 b, solid bar).

### Augmented cytotoxicity of combination of CAR T cells with iNKT cells against cancer cells expressing MSLN at a variety of intensities

**Fig. 5:**
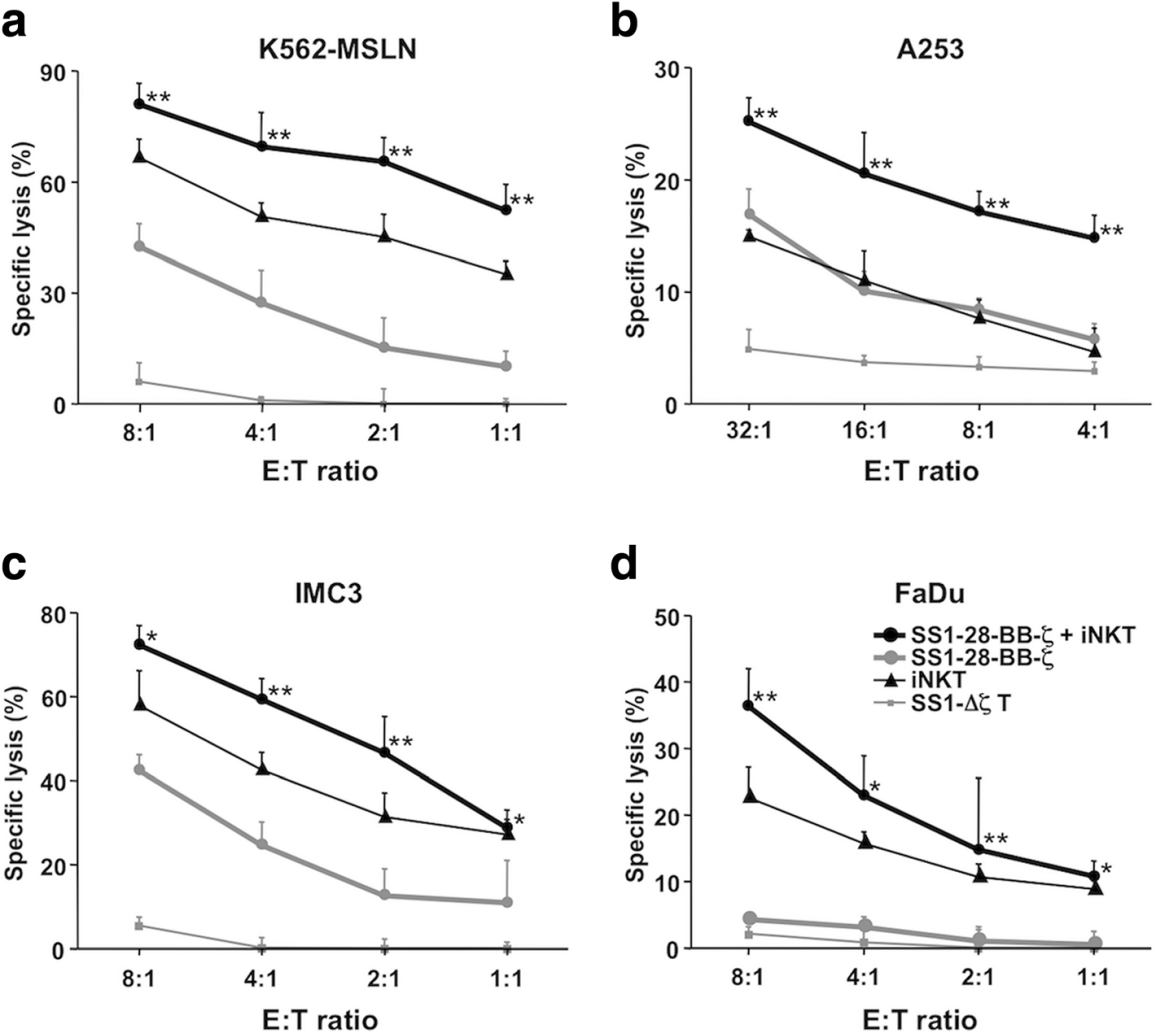
Synergistically augmented cytotoxicity of the CAR-redirected CD8 T cells and activated iNKT cells against the cancer cells expressing MSLN molecules at various intensities. Four-hour chromium-51 release assays against four kinds of target cells, MSLN-transduced K562 (**a**), A-253 (**b**), IMC-3 (**c**) and FaDu (**d**) cells, were performed. The effector cells were SS1-Δζ or SS1–28-BBζ and/or iNKT cells stimulated with αGalCer. Statistically significant differences are indicated by asterisks and daggers: *, *P* < 0.05; **, *P* < 0.01=

In a chromium-release assay with the MSLN transduced K562 target cells, SS1–28-BB-ζ CAR-transduced human CD8 T cells lysed in a dose dependent manner, and moreover, the cytotoxicity was significantly enhanced by the combination with iNKT cells (Fig. 5a). A-253 cells that moderately expressed MSLN showed the resistant to the mono-treatment with CAR-redirected T cells or iNKT cells, whereas the combined use of CAR T cells with iNKT cells resulted in significantly enhanced killing activity against A-253 cells (Fig. 5b). Further, the IMC-3 cells which mildly expressed MSLN resisted to CAR T mono-treatment, however, the combined treatment of CAR T cells with iNKT cells showed significantly augmented killing activity similar to A-253 targets. (Fig. 5c). To FaDu cells that barely expressed MSLN, the killing activity of CAR T cell alone was obscure, however the activity of the combination with iNKT cells was significantly higher than that of iNKT cell mono-treatment (Fig. 5d).

## Discussion

The chimeric antigen receptor technique provides effector T cells with high concentrations of monoclonal antigen receptors and higher affinity and avidity than conventional T cell receptors. The adoptive transfer of CAR-expressing T cells has been shown to have dramatic efficacy in patients with hematological malignancies [20, 46]; however, solid tumors are difficult to eradicate with CAR-expressing T cells. Because the target cancer antigens with sufficient affinity have not discovered, the activation and proliferation in tumor site of CAR-expressing T cells might not be enough [47]. Therefore, the development of novel adjuvant agents that enhance the anti-tumor activities of CAR T cells is critical for improving the therapeutic effects of cancer treatments.

MSLN is one of tumor antigen expressed on the various epithelial neoplasms [48]. In this study, we placed our focus on SGC that lacks effective treatments other than operation and investigated the potential of MSLN as a therapeutic target. The SGCs, which we examined, expressed MSLN except for the case of squamous cell carcinoma that is a rare in SGC. However, the expression levels varied among the surgical specimens, even within the same histological tumors, and moreover the mixtures of the cancer cells with varying expression of MSLN were observed in same sample. In order to examine the anti-tumor efficacy of MSLN-specific CAR-expressing CD8 T cells against SGC, we utilized six kinds of cancer cell lines including A-253 cells, which were derived from salivary gland carcinoma and moderately expressed MSLN.

MSLN-specific third-generation CAR T cells were activated by and proliferated in response to the stimulation of cancer cells in a MSLN intensity-dependent manner. The activated CAR T cells showed significant cytotoxic effects against A-253 cells, however the effects were limited. Interestingly, the co-culture with iNKT cells could increase the activation as well as proliferation of CAR T cells and clearly enhanced the cytotoxicity. These amplifying effects of iNKT cells were detected in various tumor cells regardless of the expression level of MSLN. Especially, in the case of mono-treatment with CAR T cells against FaDu which barely expressed MSLN, although the CAR T cells certainly showed the activation and proliferation after the exposure to FaDu cells, any effective cytotoxic properties were not confirmed. However, the co-culture with iNKT cells significantly enhanced the activation and induced cytotoxicity of CAR T cells. In this study, iNKT cells could be expected to participate in the augmentation of the signal transduction and activations of MSLN specific CAR T cells after the MSLN recognition, followed by the enhanced cytotoxicity of CAR T cells. Because iNKT cells are known to be sensitive for a wide variety of tumor cells, the combination of CAR T cells with iNKT cells may be adapted for the cancer that weakly express the target molecule.

Although the detailed mechanisms of the enhanced cytotoxicity by the co-culture of CAR T cells with iNKT cells remain to be clarified, the IFNγ produced from iNKT cells appears to play a critical role in this augmentation [49], as the treatment with anti-IFNγ antibodies abrogated the potentiation. These results suggest that combination immunotherapy with CAR-expressing CD8 T cells and iNKT cells may be effective against cancer cells that express target molecules at low levels.

## Conclusions

Further detailed in vivo studies need to be conducted to demonstrate the adjuvant effects of combined treatment of MSLN specific CAR T cells with iNKT cells and confirm the feasibility of this therapy. In addition, the adjuvant effects of iNKT cells may not be restricted to CAR T cells, but also be applicable to all forms of antigen-specific immunotherapy regimens containing antibody therapy.

## Additional file

Additional file 1: **Figure S1.** Mean fluorescence intensities of MSLN expression on the various cancer cell lines. The mean fluorescence intensities shown in Fig. 1 (h) were calculated. The light gray bars indicate isotype matched controls, the dark gray bars indicate wild type cell lines and solid bar indicates MSLM transduced K562 cells. (TIFF 8911 kb)

## Abbreviations

7AAD: 7-aminoactinomycin D
APC: Antigen-presenting cell
CAR: Chimeric antigen receptor
CTL: Cytotoxic T lymphocyte
DC: Dendritic cells
FBS: Fetal bovine serum
HNSCC: Head and neck squamous cell carcinoma
IFNγ: Interferon-γ
IMRT: Intensity modulated radiation therapy
iNKT cell: Invariant natural killer T cell
MHC: Major histocompatibility complex
MSLN: Mesothelin
NK cell: Natural killer cell
PBMC: Peripheral mononuclear cells
SCC: Squamous cell carcinoma
SGC: Salivary gland cancers
αGalCer: α-galactosylceramide
