# Supplementary figures and images for: Activated iNKT cells enhance the anti-tumor effect of antigen specific CD8 T cells on mesothelin-expressing salivary gland cancer
Source: BMC Cancer. 2018 Dec 17;18:1254. doi: 10.1186/s12885-018-5179-7 (PMC6296067; doi:10.1186/s12885-018-5179-7)

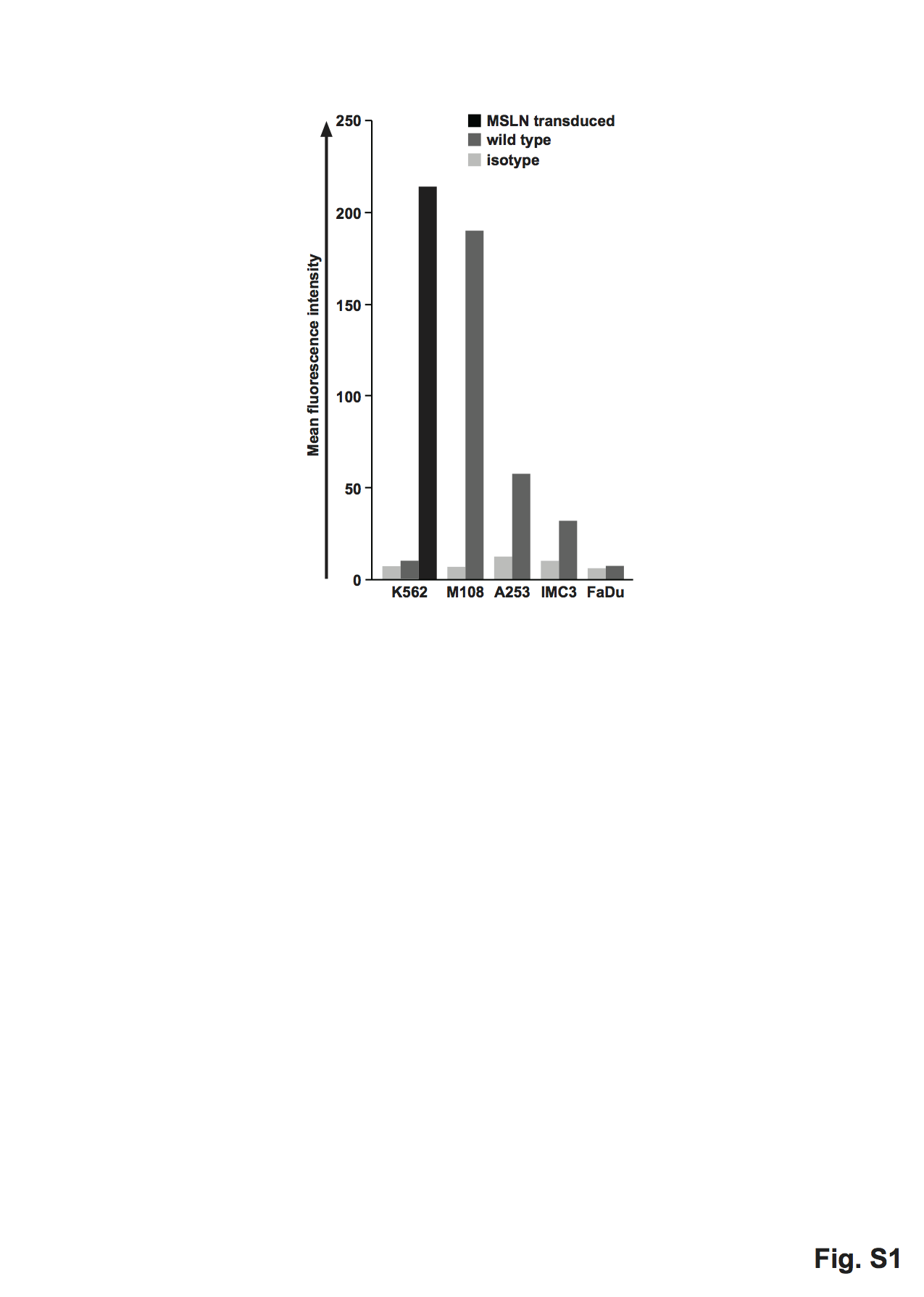

Supplement: Supplementary file 1 — Figure S1. Mean fluorescence intensities of MSLN expression on the various cancer cell lines. The mean fluorescence intensities shown in Fig. 1 (h) were calculated. The light gray bars indicate isotype matched controls, the dark gray bars indicate wild type cell lines and solid bar indicates MSLM transduced K562 cells. (TIFF 8911 kb) [file 12885_2018_5179_MOESM1_ESM.tiff]
